# Annotations

**Published:** 1901-03-23

**Authors:** 


### Annotations.

### Water London and the Housing Problem.

Among the various problems on the solution of which much of the future prosperity of London depends, two of great importance stand prominently before the public eye, namely, the water question and the housing question. They are by no means simple matters either of them, and their complexity is increased rather than diminished by the fact that they are mutually inter-dependent. If London were to attempt the hopeless task of housing its vast and growing population within its already overcrowded area there might be some excuse for the London County Council endeavouring to obtain exclusive control over the water supply, but to those who look to a large outspreading of the population and the work of London over the suburban area as the only true solution of the housing problem, the suggestion that the whole control of the water supply, upon which the development of the outer ring must depend, should be given into the hands of those who dwell within the boundary of the county and whose interests are entirely inside, hardly commends itself. No one seems to have the boldness to suggest that the London boundaries ought to be extended, but it is obvious enough that with London boroughs poaching upon each other's land in search of sites for workmen's dwellings and the County Council building new towns in surrounding counties, endeavouring to make itself master of the water supply for wide areas extending far beyond its own geographical limits, and anxious to run tramways in every direction, the time is coming when these conflicting interests should be done away with and the whole of the outer ring should be brought into the County of London. In everything we do we are more or less tied down by the doings and the customs of our ancestors, and in dividing up the country into sanitary areas, all considerations of water-shed, water supply, and drainage had to be put on one side in order that the sanitary areas might coincide with pre-existing divisions which were sanctioned by custom and tradition. But London might well be made an exception to this general rule. Undoubtedly there should be one central supervising authority which should have the control over the water supply, the drainage, and the buildings, and should fix the standard of sanitation, not merely over the limited area which is included within the boundaries of the County of London, but over that far larger area which is inhabited by London people. We have no wish to see set up an *imperium in imperio* such as might arise were the powers of the County Council, as at present constituted, extended over so large a portion of the home counties as this would involve, but there are certain things in regard to which there must be co-operation and community of action between London and the surrounding districts. These things are water supply, drainage, and standard of sanitation—including, of course, the permissible standard of house-building—and these things might well be relegated to some central body. At the present time notwithstanding all that is being said about slum dwellings and unhealthy areas there is much reason to fear that London is allowing itself to be built in by a whole circle of slum suburbs, the water supply of which is dependent upon the water companies happening to have a surplus, the drainage of which goes much as it listeth, and the buildings and streets of which are obviously below the standard set up by the London Building Act. Sometime or other all these suburbs will have to be absorbed, and then again London will be faced with the problem what to do with unhealthy areas the growth of which is now going on under our very eyes.

### The German Army Medical Service.

Now that the Army Medical Department, and indeed all army departments, are about to be thrown into the melting pot, it is worth our while to consider how our good friends the Germans, who are a practical people and thrifty withal, set about the task of obtaining medical officers for their vast army. As in every little detail in their military organisation, so in obtaining medical recruits, they are vastly aided by their system of compulsory military service. If a man does not serve one way he must another; and as most men of education would give their ears to escape becoming privates, there is quite a run on the medical service. The point, however, to which we would direct special attention is that, instead of going into the market as it were and engaging medical men just when they are freshly qualified, just when they are hoping at last to see some return for all their education has cost them, and when not unnaturally their estimate of their own value is at its highest, being as yet untempered by experience, the German Government to a very considerable extent educates its own medical officers from the beginning. It is at the moment when the fond but, may be, impecunious parent of the promising boy is wondering what to do with him, and when the youth is fearing greatly that an office stool will be his lot in life, that the State steps in, and on condition that he will undertake to enter the Army Medical Service offers to send him to a medical school, to give him lodging during the time that he is there, and to provide him with a certain small amount of pay. The Kaiser Wilhelm Academie in Berlin has about 200 students, chosen after competitive examination, all of whom are in training as military surgeons. They receive from the State free lodging and tuition and 30 marks monthly, while for necessary medical books, preparations and instruments, the student pays one-third and the State two-thirds. After they have completed their regular course they have a year of hospital service in Berlin, and are then assigned to duty as assistant surgeons. After some years of this work they come back for post-graduate study, and if they have distinguished themselves many facilities are put in their way for joining the staffs of the civil hospitals. They still, however, remain in a manner connected with the army, the State having a lien on their services in case of necessity. In actual working this method gives to the State many great advantages in return for a comparatively small outlay. It not only gets a number of first-class medical officers at a comparatively cheap rate, but it gradually develops a medical reserve which is not only numerous but contains within its ranks the very cream of the profession—consultants, specialists, and professors—men actively engaged in professional work, and yet ready to join and take up their positions in the army at a moment's notice should their services be required. We do not say that such a system is a benefit to the medical profession—probably it is quite the opposite, seeing that it artificially increases the number of doctors —but it is a highly practical and very thrifty proceeding from the State point of view.

### Professional Reticence.

The sympathy of all good men will be accorded to the two house surgeons of the East Suffolk Hospital, Ipswich, who have been dismissed in consequence of their refusal to fill out and sign a stupid form which it seems to have been the custom to supply to the local press giving particulars about any cases of accident which might be admitted to that institution. The attempt to impose such a duty upon the house surgeons is but another, and a somewhat flagrant, example of the laxity with which some men regard the obligation not to place one's signature to anything which one does not know of one's own knowledge. In addition to stating the nature of the accident, and thus making themselves a party to the publication of knowledge in regard to their patient which has come to them purely from their professional relation to him, these house surgeons are asked to state, over their signatures, a series of particulars which they can have no possible means of knowing except from hearsay, all of which is absolutely wrong. That in his ordinary duties a house surgeon, for the guidance of the surgeons, or as a necessary means of keeping in touch with the friends and relatives of the patient, should obtain such particulars and should enter them in a book would be right and proper. All this would be done for the patient's benefit, and any such record would no doubt be regarded as privileged were any legal process to result. Quite otherwise, however, might it be were a house surgeon to go out of his way to give such information to the representatives of the press. We go back, however, to our first point. We think that it is wrong that the house surgeons should be asked to publish any information about their patients which they could only obtain in their professional capacity. We hold that, so far as is consistent with the orderly management of a hospital, hospital patients are entitled to the same consideration as private patients are. If the secretary of a hospital chooses to place the hospital admission-book at the disposal of the press, we should perhaps regard it as a regrettable circumstance, but should feel that it was outside medical control. But to ask a medical man to give details regarding his hospital patient which he certainly would not feel at liberty to give in regard to any private case, is not only wrong from a professional point of view, but is, we think, a manner of dealing with hospital patients which even charity will not cover or excuse.

### The Polyclinic.

The continued success of the Medical Graduates' College and Polyclinic is a matter on which those to whose energy its remarkable progress is due deserve sincerest congratulation. According to the report which will be presented to the annual meeting next Tuesday everything except the one thing needful, that is money, is in an absolutely flourishing condition, and we can hardly believe that those who have the means at their disposal will allow an institution which has shown such vitality in regard to all that concerns teaching, and has so usefully opened up quite a new departure in medical charity, to suffer for want of a share of that superabundance of wealth which is ever seeking outlets through which it may do good to humanity. So far as teaching is concerned it appears that the College has arranged a series of practical classes, the subjects selected being those in regard to which the practitioners attending show most desire to renew their knowledge; laboratory classes are also at work; clinical lectures by well-known teachers are regularly given; extramural classes are arranged for, and a system of hospital practice in association with the College has been organised. The charitable work of the College lies, however, in the consultations, which are held every afternoon at 4 o'clock, at which patients are examined, their symptoms are discussed, and, if desired, an opinion is forwarded to the medical man tending the case. These afternoon consultations have proved to be of very great interest to the practitioners attending them, and no one can doubt that they offer a great boon to an enormous multitude of sick folk who are as anxious as their wealthier neighbours for a "second opinion" but are entirely unable to pay for such a luxury. At the Polyclinic the doctor in attendance can go up with his own patient and take part in the consultation, or he can send up his patient in full confidence that he will receive a careful opinion upon the case. This is a departure in medical charity which must appeal to many as giving great help and comfort to a large and highly deserving class of sufferers, and it is to be hoped that this charitable side of the work of the Polyclinic will receive substantial recognition at the Annual Dinner on May 22nd, at which the Right Hon. A. J. Balfour will preside. Upwards of a thousand consultations were held at the Polyclinic during the year 1900.

### Precautions against Plague.

At the meeting of the Association of Port Sanitary Authorities held last week at Hull, a paper was read by Dr. Robinson, of Dover, on "The Recognition of Plague," in which it was pointed out that recent experience had shown that the aid of the bacteriologist was necessary to establish a proper diagnosis of the disease, and to clear up any doubt in its history. Dr. Mason, of Hull, also read a paper on "Recent Experiences of Plague in Hull." In the discussion which followed, emphasis was laid upon the necessity for supervising the discharge of cargoes of vessels from infected or suspected ports, it being pointed out that vessels having plague rats on board should be treated as plague-infected ships. A resolution was passed that the Association, recognising the great danger associated with the discharge of vessels infested by rats, recommend the Local Government Board to alter the law so as to strengthen the hands of port sanitary authorities in such matters.

